# A mutation in the methionine aminopeptidase gene provides phage resistance in *Streptococcus thermophilus*

**DOI:** 10.1038/s41598-019-49975-4

**Published:** 2019-09-25

**Authors:** Simon J. Labrie, Cas Mosterd, Stéphanie Loignon, Marie-Ève Dupuis, Philippe Desjardins, Geneviève M. Rousseau, Denise M. Tremblay, Dennis A. Romero, Philippe Horvath, Christophe Fremaux, Sylvain Moineau

**Affiliations:** 1 Département de biochimie, de microbiologie, et de bio-informatique, Faculté des sciences et de génie, Université Laval, Québec City, QC G1V 0A6 Canada; 2 Groupe de recherche en écologie buccale, Faculté de médecine dentaire, Université Laval, Québec City, QC G1V 0A6 Canada; 3 Félix d’Hérelle Reference Center for Bacterial Viruses, Faculté de médecine dentaire, Université Laval, Québec City, QC G1V 0A6 Canada; 4 DuPont Nutrition and Biosciences, 3329 Agriculture Dr, Madison, WI 53716 USA; 5 DuPont Nutrition and Biosciences, BP10, Dangé-Saint-Romain, 86220 France; 6 Present Address: SyntBioLab Inc., 4820-250, rue de la Pascaline, Lévis, G6W 0L9 Canada

**Keywords:** Applied microbiology, Bacteriophages

## Abstract

*Streptococcus thermophilus* is a lactic acid bacterium widely used by the dairy industry for the manufacture of yogurt and specialty cheeses. It is also a Gram-positive bacterial model to study phage-host interactions. CRISPR-Cas systems are one of the most prevalent phage resistance mechanisms in *S*. *thermophilus*. Little information is available about other host factors involved in phage replication in this food-grade streptococcal species. We used the model strain *S*. *thermophilus* SMQ-301 and its virulent phage DT1, harboring the anti-CRISPR protein AcrIIA6, to show that a host gene coding for a methionine aminopeptidase (*metAP*) is necessary for phage DT1 to complete its lytic cycle. A single mutation in *metAP* provides *S*. *thermophilus* SMQ-301 with strong resistance against phage DT1. The mutation impedes a late step of the lytic cycle since phage adsorption, DNA replication, and protein expression were not affected. When the mutated strain was complemented with the wild-type version of the gene, the phage sensitivity phenotype was restored. When this mutation was introduced into other *S*. *thermophilus* strains it provided resistance against *cos*-type (*Sfi21dt1virus* genus) phages but replication of *pac*-type (*Sfi11virus* genus) phages was not affected. The mutation in the gene coding for the MetAP induces amino acid change in a catalytic domain conserved across many bacterial species. Introducing the same mutation in *Streptococcus mutans* also provided a phage resistance phenotype, suggesting the wide-ranging importance of the host methionine aminopeptidase in phage replication.

## Introduction

Bacteriophages are one of the most important drivers of bacterial evolution as they exert a constant selective pressure^1^. Bacteria inevitably evolve to acquire phage resistance, which is often associated with a fitness cost^2–5^. This evolutionary arms race underscores the complex network of phage-host interactions^6–8^. Comprehensive knowledge of how phages interact with their host components is available for only some *Escherichia coli* phage-host systems as the literature is very sparse for other bacterial species.

To protect themselves against these viral invaders, bacterial hosts have acquired and evolved numerous phage resistance systems^6,8^. These anti-phage systems are generally classified based on the step of the infectious cycle they interfere with, for example, inhibition of phage adsorption, preventing DNA entry, DNA degradation, and abortive infection, among others^6^. However, our knowledge of the bacterial arsenal against phages is partial as new defense systems are still being discovered^9–12^.

*Streptococcus thermophilus* is a lactic acid bacterium extensively used in milk fermentation, second only to *Lactococcus lactis* in its widespread usage. Phages infecting *S*. *thermophilus* have been historically divided into two large groups, the so-called *cos-* and *pac-*type phages, also named the *Sfi21dt1virus* and *Sfi11virus* genera, respectively. This grouping is based on comparative genome analyses, DNA packaging strategy and the number of major structural proteins^13^. In 2011, *S*. *thermophilus* phage 5093, which did not share the common features of either group, was characterized, prompting the creation of the eponymous taxon^14^. Five years later, four members of a new *S*. *thermophilus* phage taxon (987) were reported^15^. Still the *cos-* and *pac*-type phages are by far the most common *S*. *thermophilus* phages worldwide and are responsible for most milk fermentation failures^16–19^.

Only a few phage resistance mechanisms have been identified in *S*. *thermophilus*. Among the most common phage defense systems encoded by *S*. *thermophilus* genomes there are restriction-modification systems, as reflected by the number of different entries in REBASE (http://rebase.neb.com). Two superinfection exclusion (Sie) mechanisms encoded by prophages were also identified in *S*. *thermophilus*^20,21^. Sie systems interfere with the phage genome ejection into the host cytoplasm. However, it is the CRISPR-Cas systems that seem to be the most dominant defense mechanism in this bacterial species^9,22^. CRISPR-Cas systems are bacterial adaptive immune systems that protect the cell from invading nucleic acids via targeted cleavage^23^. The bacterial cell accumulates short sequences, named spacers, from the phage genomes into CRISPR arrays that are composed of these spacers interspersed by short repeats^9,24^. These spacers act as the cell’s memory of previous encounters and serve as guides for specific cleavage of invading DNA. The adaptive nature and efficiency of this system may explain why only a few other phage resistance mechanisms have been identified in *S*. *thermophilus*. However, the recent discovery of anti-CRISPR proteins (AcrIIA5 and AcrIIA6) in *S*. *thermophilus* virulent phages illustrates the ongoing armsrace between phages and their hosts, and suggests that additional mechanisms are at play^25,26^.

Recently, phage-immunity systems in *S*. *thermophilus* that are linked to cryptic, non-inducible prophages were discovered^11^. Another study demonstrated that transient inactivation of the CRISPR-Cas system allows isolation of non-CRISPR-mediated bacteriophage insensitive mutants (BIMs)^27^. Characterization of these non-CRISPR BIMs offers the possibility of identifying new bacterial factors that can be mutated to reduce phage infection such as the newly described mutations in cell wall glycans^28^.

Here, we used the *cos*-type virulent phage DT1^29^ and its host strain SMQ-301 to identify an additional host factor involved in phage infection. This phage encodes an anti-CRISPR protein which is naturally disabling most of the CRISPR1-Cas system of *S*. *thermophilus*^26^, one of the two type II-A systems active in *S*. *thermophilus* SMQ-301. Using this phage-host system, spontaneous BIMs selected after phage challenge either arise from acquisition of new spacer within the CRISPR3 array^24,30^ or from unknown mechanism(s). Sequencing the genome of *S*. *thermophilus* SMQ-301 BIMs that had not acquired new spacers led to the identification of the methionine aminopeptidase (MetAP) as a key host factor for the replication of *S*. *thermophilus cos*-type phages.

## Materials and Methods

### Bacterial growth and phage propagation

Bacterial strains, phages and plasmids used in this study are listed in Table 1 and Table 2. *S*. *thermophilus* was grown at 37 °C for the pre-cultures or 42 °C for the assays in M17 medium (Oxoid) supplemented with 0.5% lactose (LM17). When needed, chloramphenicol (Sigma) was added to a final concentration of 5 µg/mL for growth and selection of *S*. *thermophilus* strains containing pNZ123^31^ and derivatives. Agar was added to a final concentration of 1% for solid media. *Escherichia coli* was grown with agitation at 37 °C in Luria Broth (LB) supplemented with 20 µg/mL chloramphenicol for selection. *Streptococcus mutans* HER 1503 was grown at 37 °C and 5% CO_2_ in Brain Heart Infusion (BHI, Difco), supplemented with 10 µg/mL of chloramphenicol when selecting for cells carrying pNZ123. Phages were propagated as previously described^32^. Phage adsorption assays were performed as described elsewhere^33^ and phage DNA replication assays were performed as reported^34^.

**Table 1:** Plasmids and bacterial strains used in this study.

Name	Characteristics	Reference
Plasmids		
pNZ123	*E*. *coli*, *L*. *lactis* and *S*. *thermophilus* shuttle vector	^31^
pNZ123:*metAP*	pNZ123 with *metAP* gene cloned in XbaI site	This study
pNZ123:*metAP*^H206Q^	pNZ123 with *metAP*^*H206Q*^ gene cloned in XbaI site	This study
pNZ123:*metAP*Smut	pNZ123 with *metAP* of *S*. *mutans* HER1503 gene cloned in XbaI site	This study
Bacterial strains		
*S*. *thermophilus* SMQ-301		^36^
*S*. *thermophilus* SMQ-301 BIM #2		This study
*S*. *thermophilus* SMQ-301 BIM #3		This study
*S*. *thermophilus* SMQ-301 BIM #5		This study
*S*. *thermophilus* SMQ-301:*metAP*^H206Q^		This study
*S*. *thermophilus* SMQ-301:*metAP*^H206Q^ + pNZ123: *metAP*		This study
*S*. *thermophilus* DGCC7710		^9^
*S*. *thermophilus* DGCC7710:*metAP*^H206Q^		This study
*S*. *thermophilus* DGCC7710:*metAP*^H206Q^ + pNZ123: *metAP*		This study
*S*. *thermophilus* DGCC7796		This study
*S*. *thermophilus* DGCC7796:*metAP*^H206Q^		This study
*S*. *thermophilus* DGCC7796:*metAP*^H206Q^ + pNZ123:*metAP*		This study
*S*. *thermophilus* DGCC782		This study
*S*. *thermophilus* DGCC782:*metAP*^H206Q^		This study
*S*. *thermophilus* DGCC782:*metAP*^H206Q^ + pNZ123:*metAP*		This study
*S*. *mutans* HER 1503		^43^
*S*. *mutans* HER 1503:*metAP*^H206Q^		This study
*S*. *mutans* HER 1503:*metAP*^H206Q^ + pNZ123:*metAP*Smut		This study

**Table 2:** Phages used in this study.

Phages	Host range					Ref.
	*S*. *thermophilus* SMQ-301	*S*. *thermophilus* DGCC7796	*S*. *thermophilus* DGCC7710	*S*. *thermophilus* DGCC782	*S*. *mutans* HER 1503	
*S*. *thermophilus cos-*type phages						
DT1	+					^29^
MD2	+					^47^
D4090		+				This study
D4807		+				This study
D5821		+				This study
D5691			+			This study
D5913			+			This study
D6037			+			This study
D6215			+			This study
N1032				+		This study
N1117				+		This study
N1119				+		This study
N1169				+		This study
N1358				+		This study
N3782				+		This study
*S*. *thermophilus pac-*type phages						
D2765		+				This study
D4274		+				This study
M5876		+				This study
D5787		+				This study
858			+			^9^
2972			+			^48^
D4259			+			This study
D939			+			This study
D3288			+			This study
D4752			+			This study
D4754			+			This study
*S*. *mutans cos*-type phage						
M102AD					+	^43^

### Bacteriophage-insensitive mutant isolation

BIMs were isolated using the soft agar overlay assay as detailed elsewhere^32^. Briefly, the wild-type host SMQ-301 was challenged with the lytic phage DT1. The bacterial host was grown in LM17 at 42 °C until the optical density at 600 nm (OD_600nm_) reached 0.6. Then, 300 µL of the culture was added to 3 mL of molten LM17 soft agar supplemented with calcium chloride (10 mM) and phage DT1 was added to achieve a multiplicity of infection (MOI) of 0.1. Plates were incubated at 42 °C for 16 h. The resulting colonies were streaked on LM17 + agar and screened for spacer acquisition in CR1 and CR3 loci using primers CR1-fwd and CR1-revLong for the CR1 locus, and primers CR3-fwd and CR3-rev for the CR3 locus^32^ (Table S1). When spacer acquisition was not detected in CRISPR arrays, BIMs were conserved for genome sequencing.

### DNA isolation, sequencing and bioinformatics analysis

The genomic DNA of the selected BIMs was extracted as previously described^35^. The genomes were sequenced using the Illumina MiSeq platform. The libraries were prepared with the Nextera XT DNA sample preparation kit according to the manufacturer’s instructions and sequenced using MiSeq reagents (2 × 250 nt paired-end). The average coverage ranged from 6.8- to 80.1-fold. The DNA reads obtained for the genome of nine BIMs were aligned on the *S*. *thermophilus* SMQ-301 wild-type genome^36^ using Novoalign (http://www.novocraft.com) with the default settings. The mutations were extracted from the alignment file using SAMTools^37^. In-house Python scripts were used to map the mutations in the bacterial genome and to determine their impact in coding sequences. The mutations with the highest score as provided by SAMTools were considered first.

### Complementation assays

The BIM *S*. *thermophilus* SMQ-301:metAP^H206Q^ was complemented with the wild-type *metAP*. First, *metAP* was cloned into pNZ123 using Gibson assembly^38^. The vector pNZ123 was linearized with XbaI (Roche) according to the manufacturer’s instructions. The insert was amplified with the primers SJL154 and SJL155 (Table S1) with Q5 high-fidelity DNA polymerase (NEB). Both primers had 30-nt extensions complementing the 3′- and 5′-ends, respectively, of the linearized vector. An insert/vector ratio of 3:1 was used for assembly. The master mixture for Gibson assembly was prepared as described previously^38^. The resulting construction was transformed into *E*. *coli* NEB5α according to the manufacturer’s instructions and the clones were selected on LB medium supplemented with agar and 20 µg/mL chloramphenicol. One clone was confirmed by Sanger sequencing (ABI 3730xl) at the Plateforme de séquençage et de génotypage des génomes at the Université Laval. Plasmid DNA was extracted using a QIAprep Spin Miniprep kit (Qiagen) and transformed into *S*. *thermophilus*^32^. The clones were selected by spreading the transformation mixture on LM17 supplemented with agar and 5 µg/mL of chloramphenicol.

### Proteomic analysis of the phage-infected *S*. *thermophilus* DGCC7796 cells

Phage infection and protein analyses were performed as previously described^39^. Briefly, *S*. *thermophilus* DGCC7796 and *S*. *thermophilus* DGCC7796:metAP^H206Q^ were each grown to an OD_600nm_ of 0.5 at 42 °C. The cultures were infected with *cos*-type phage D4090 or *pac*-type phage M5876 at a MOI of 5. The infections were arrested after 20 min by harvesting the infected cells by centrifugation and the cell pellet was flash-frozen at −80 °C. The cell pellet was quickly thawed and resuspended in 200 µL of lysis buffer (0.5% sodium deoxycholate, 50 mM ammonium bicarbonate, 5 mM DL-Dithiothreitol (DTT), Complete Protease Inhibitor Cocktail (1 µL for 12.5 mL of buffer, Roche)). An equal volume of acid-washed glass beads was added to the cell suspension. The mixture was vortexed in a Mini-Beadbeater-8 cell (BioSpec Products) five times for 1 min with 1 min intervals on ice. The final lysate was centrifuged and the soluble fraction was kept at −80 °C until analysis. The LC-MS/MS analysis was done using a QTRAP 6500 at the Centre de Protéomique de l′Est du Québec. The spectra were analyzed using the complete peptide database, a peptide database that contained only the N-terminal peptides, and a database that contained only the N-terminal peptides without the N-terminal methionine. The latter two databases allowed the distinction of post-translational processed proteins from native proteins that still had the N-terminal methionine. The final analysis was done with Scaffold v4.

### Directed and random mutagenesis

The *metAP* gene with the desired mutation was amplified by PCR using the primers SJL128 and SJL130 (Table S1). At least 10 PCR reactions of 50 µL were done for each assay to obtain enough DNA for natural transformation. All reactions were pooled and precipitated by adding 1.5 mL of 95% ethanol and 75 µL of sodium acetate 3 M (pH 5.2). The tubes were centrifuged at 25,000 × g for 20 min at 4 °C. The DNA pellet was washed twice with 1 mL of 70% ethanol and let dry for 10 min at room temperature to remove traces of ethanol. Finally, the pellets were dissolved in 150 µL of water. The linear DNA fragments were introduced into *S*. *thermophilus* by natural transformation^40,41^. Briefly, *S*. *thermophilus* strains SMQ301, DGCC7710, DGCC7796 and DGCC782 were grown overnight at 37 °C in 1 mL of LM17. The cultures were centrifuged at 17,000 × g for 1 min, and the bacterial cells were washed twice with chemically defined medium (CDM)^40,41^ and recovered in 1 mL of CDM. The final cultures were diluted 30-fold to obtain an OD_600nm_ of 0.05 and incubated 75 min at 37 °C before storing at −20 °C. For the transformation, 1 µM of the ComS peptide and 1 µg of linear DNA were added to 300 µL of naturally competent cells. The mixture was incubated for 3 h at 37 °C. The cells were serially diluted and spread on non-selective LM17 agar to obtain isolated colonies. The colonies were screened using a PCR approach specifically designed to detect clones with the desired mutation. One of the primers used in this protocol included the desired mutation at its 3′-end. Thus, the PCR amplification was only positive if the mutation was present in the clone screened. Primers SJL150 and SJL151 (Table S1) were used for screening the transformants. A similar approach was used for random mutagenesis, but we amplified the wild-type *metAP* gene using an error-prone PCR amplification. The proof-reading activity of the DNA polymerase was reduced by adding 10 µM of MnSO_4._. Phage DT1 was used for the selection of resistant clones.

### Directed *metAP* mutagenesis in *Streptococcus mutans*

The *metAP* gene was amplified from *S*. *mutans* HER 1503 with two sets of primers containing the desired mutation (MetAP^H206Q^). The two sets of primers (SJL160 and SJL161 as well as SJL162 and SJL163; Table S1) generated two overlapping amplicons, with the mutation in the overlapping region. The two fragments were purified using the QIAquick PCR purification kit (Qiagen) and fused by Gibson Assembly^38^. A PCR was then performed on the Gibson Assembly sample using primers SJL160 and SJL163 (Table S1) to amplify the fused fragments. After purification using the QIAquick PCR purification kit, the mutated *metAP* gene was transformed into *S*. *mutans* HER 1503 by natural transformation. The Competence Stimulating Peptide (CSP) ordered from Biomatik was added to 500 μl of an exponentially growing culture of *S*. *mutans* (OD_600nm_ of 0.1) at a concentration of 1 μM along with 1 μg of purified mutated *metAP* amplicon^42^. The culture was incubated overnight at 37 °C with 5% CO_2_, then mixed with the virulent phage M102AD^43^ in BHI supplemented with 0.7% agar and plated onto BHI supplemented with agar. The *metAP* gene in the surviving BIMs was amplified by PCR using primers SJL160 and SJL163 (Table S1) and sequenced to confirm the mutation.

For the complementation of the *S*. *mutans* BIM HER 1503:metAP^H206Q^, the wild-type *S*. *mutans metAP* was cloned into pNZ123. pNZ123 was linearized with XhoI and EcoRI to remove the 24 base pairs between the two sites and the resulting plasmid was purified using the QIAquick Gel Extraction kit (Qiagen). The *metAP* gene was amplified from *S*. *mutans* HER 1503 using primers CM145 and CM146 (Table S1) to generate the gene, flanked by XhoI and EcoRI restriction sites. The PCR sample was purified using the QIAgen PCR purification kit, cut by XhoI and EcoRI and ligated into the linearized pNZ123 using T4 DNA ligase. The molar insert/vector ratio used during the ligation was 3:1. The ligated plasmid was transformed into *E*. *coli* NEB5α and plated onto solid LB medium supplemented with 20 µg/mL chloramphenicol. Amplification of the *metAP* gene by primers CM145 and CM146 and sequencing confirmed the sequence cloned in the complementation plasmid. Plasmid DNA was extracted using a QIAprep Spin Miniprep kit and transformed into the *S*. *mutans* HER 1503:metAP^H206Q^. The strain was grown in BHI until an OD_600nm_ of 0.1 was reached, an aliquot of 500 µL was exposed to 1 µM CSP and transformed with 1 µg of the complementation plasmid. After a 2.5-hour incubation period at 37 °C with 5% CO_2_, the culture was plated onto BHI + agar supplemented with 10 µg/mL of chloramphenicol.

### Growth curves of *S*. *thermophilus* strains

Bacterial strains were grown overnight at 37 °C. M17 medium (Nutri-Bact) supplemented with 0.5% lactose (LM17) were inoculated at 1% with the pre-cultures and incubated at 42 °C. The OD_600nm_ was taken every 30 min for 8 hours. Generation time was calculated based on three biological triplicates and from the linear section of the semi-logarithm graph of the OD_600nm_ as a function of time.

### Mutation stability test

To test the stability of the mutation H206Q, we inoculated (1%) the strains *S*. *thermophilus* SMQ-301 and DGCC7796 and their respective mutants in 10 mL of M17 medium (Nutri-Bact) and in reconstituted milk (10% nonfat dry milk). The strains were incubated at 42 °C during the day. Then, an inoculum of 1% was transferred to fresh media or milk and incubated at 37 °C for the night. After 9 transfers (5 days, 4 nights) corresponding to approximately 60 generations, we randomly selected 10 colonies from each of the four strains and tested them for sensitivity to phage DT1 (*cos*) for *S*. *thermophilus* SMQ-301 and *S*. *thermophilus* SMQ-301:metAP^H206Q^, and phages D4090 (*cos*) and D4274 (*pac*) for *S*. *thermophilus* DGCC7796 and *S*. *thermophilus* DGCC7796:metAP^H206Q^. Sequencing of the *metAP* gene and CR1 was done for SMQ-301 and SMQ-301:metAP^H206Q^ colonies with primers SJL128/SJL130 and CR1-fwd/CR1-revLong, respectively, to confirm both the presence of the mutation after the transfers and the identity of the strains.

## Results and Discussion

### A mutation in the gene coding for the methionine aminopeptidase provides phage resistance

**Figure 1:**
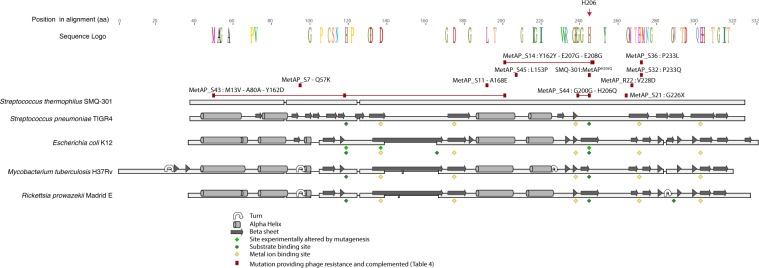
Protein alignment of *S*. *thermophilus* SMQ-301 MetAP with 4 other MetAP for which the structure is available. The WebLogo was only kept when the residue was conserved in all sequences. The secondary structures are represented over each sequence and diamonds indicate active sites and substrate binding sites. Red boxes highlight the mutations that provide phage resistance listed in Table 4. When linked, they occurred in the same MetAP mutant of *S*. *thermophilus* SMQ-301. Uniprot accession number of the protein sequences: *E. coli* K12 (P0AE18), *Rickettsia prowazekii* Madrid E (Q9ZCD3), *S. pneumonia* TIGR4 (B2IQ22) and *M. tuberculosi*s H37Rv (P9WK19).

We isolated BIMs of *S*. *thermophilus* SMQ-301 that were resistant to phage DT1. Most of the BIMs had acquired new spacers targeting the phage DT1 genome in their CRISPR3 locus (data not shown). Since we were interested in the discovery of other host factors involved in phage infection, we selected nine SMQ-301 BIMs that had not acquired any new spacer, conjecturing they had a mutation elsewhere in their chromosome to provide phage resistance. We sequenced the genomes of these nine non-CRISPR BIMs. By comparing these sequences with that of the wild-type strain SMQ-301 (accession CP011217.1)^36^ we found several mutations (data not shown). Although many of these mutations may potentially be involved in phage infection, the same transversion at position 1,426,980 occurred in the genomes of three of the nine BIMs (designated BIM #2, #3, and #5). This mutation resides within the gene coding for methionine aminopeptidase (MetAP; locus_tag SMQ301_1544, T618G) and induces an amino acid substitution from a histidine to a glutamine at position 206 (H206Q) in the protein. BLAST searches indicated that this histidine is conserved in all MetAP sequences analyzed, including those deduced from all *S*. *thermophilus* sequences currently available in GenBank (29 complete and 27 draft genomes; data not shown). While the MetAP of *S*. *thermophilus* has not been previously studied, it shares 89% identity and 96% similarity with the MetAP of *Streptococcus pneumoniae* TIGR4 for which the structure has been determined (PDB 4KM3)^44^. Although the histidine at position 206 was not annotated as part of the active site (Fig. 1), this residue was shown to be a substrate-binding site in the MetAP of *E*. *coli*^45^. While this site is not essential for the catalytic activity of *E*. *coli* MetAP, replacing the corresponding histidine at position 178 with an alanine led to an impairment of the catalytic activity by 70–190 fold^46^.

### A mutation in the *metAP* gene confers phage resistance in *S. thermophilus*

To confirm that the phage resistance phenotype was due to the MetAP^H206Q^ mutation – and not to other mutations in the genome – the mutated gene *metAP*^H206Q^ was amplified and transformed into wild-type strain SMQ-301 using natural competence. Selecting with phage DT1 is very efficient, but it also increases the odds of selecting CRISPR BIMs as well as other mutations that confer phage resistance. Instead, we designed a PCR strategy to detect the transformants that integrated the PCR product with the mutation into their genome, in the absence of phage selective pressure. We used the primers SJL150 in combination with SJL151 with the desired mutation at its 3′-end (Table S1) to specifically detect the mutation in the bacterial genome. To avoid false positives due to the potential presence of residual linear DNA from the transformation inside the bacterial cytoplasm, we designed the primer SJL150 that matched the flanking genomic region of the transformed DNA fragment. A total of 282 clones were screened for the desired mutation and four positive clones were obtained (MetAP^H206Q^). We randomly selected one of these clones (designated SMQ-301:metAP^H206Q^) and tested its phage resistance phenotype. No plaque was visible on the bacterial lawn of SMQ-301:metAP^H206Q^ using various titers of the phage DT1, indicating a very strong phage resistance phenotype (Table 3). It was possible to observe a lysis zone at high phage titers, likely due to lysis from without or to the endolysin activity in the phage lysate. We also tried to isolate DT1 phage mutants that would overcome the effect of the MetAP^H206Q^ mutation using various experimental conditions, but to no avail. We examined incubation temperature, anaerobic conditions, added different concentrations of glycine to the medium to weaken the bacterial cell wall, replaced agar with different concentrations of agarose and even using liquid medium in case the lysis plaque were not visible enough. Overall, we were unable to obtain phage DT1-derivatives able to propagate on SMQ-301:metAP^H206Q^.

**Table 3:** Effect of MetAP mutations on phage efficiency of plaquing (EOP) and adsorption.

	Phage	Strain + pNZ123		Strain + pNZ123:*metAP* or pNZ123:*metAPSmut*	
		*EOP*	*Adsorption*(*%*)	*EOP*	*Adsorption*(*%*)
*S*. *thermophilus* SMQ-301	DT1	1	93.8 ± 2.2	1	90.9 ± 3.3
*S*. *thermophilus* SMQ-301 BIM #2	DT1	1.7 × 10^−8^	86.2 ± 3.2	1.7	86.0 ± 2.1
*S*. *thermophilus* SMQ-301 BIM #3	DT1	1.7 × 10^−8^	83.2 ± 7.1	0.02	89.1 ± 2.0
*S*. *thermophilus* SMQ-301 BIM #5	DT1	1.7 × 10^−8^	80.4 ± 7.7	2.4	89.0 ± 4.4
*S*. *thermophilus* SMQ-301:metAP^H206Q^	DT1	1.7 × 10^−8^	68.0 ± 3.8	1	94.9 ± 1.1
*S*. *mutans* HER 1503	M102AD	1	ND	ND	ND
*S*. *mutans* HER 1503:metAP^H206Q^	M102AD	<1 × 10^−7^	ND	1.1	ND

ND = Not determined; DT1 initial titer = 6 × 10^9^ PFU/ml; M102AD initial titer = 1 × 10^9^ PFU/ml. PFU = Plaque forming unit.

### Complementation with the wild-type allele restores phage sensitivity

We cloned the wild-type *metAP* gene from strain SMQ-301 into the expression vector pNZ123. The resulting construct was transformed into *S*. *thermophilus* SMQ-301:metAP^H206Q^ to complement the mutation in *trans*. Sensitivity to phage DT1 was completely restored for the two of the three spontaneous BIMs complemented with plasmid pNZ123:metAP (Table 3), confirming that the metAP^H206Q^ mutation was solely responsible for the phage resistance phenotype. Of note, the sensitivity to phage DT1 was not completely restored (EOP = 0.02) for the spontaneous BIM #3, suggesting that other mutations are at play (Table 3). The mutated SMQ-301:metAP^H206Q^ *metAP* gene was also cloned into pNZ123 and transformed into the wild-type strain SMQ-301. There was no difference in EOP between the strain with pNZ123 when compared to the strain with pNZ123:metAP^H206Q^, suggesting that the mutation does not produce a dominant phenotype.

### The MetAP mutation has a broad range of action against *cos*-type phages

To determine if the resistance provided by the MetAP^H206Q^ mutation is phage-dependent, we tested the *cos*-type phage MD2^47^, which is also capable of infecting strain SMQ-301. Phage MD2 was also severely inhibited by the mutated MetAP (EOP <1 × 10^−6^). To show that this mutation is not bacterial strain-dependent, we introduced the MetAP^H206Q^ mutation into several *S*. *thermophilus* strains using directed mutagenesis. We introduced the mutation into strain DGCC782, which is sensitive to *cos*-type phages N1032, N1117, N1119, N1169, N1358 and N3782 (Table 2). The mutation was also introduced into the strain *S*. *thermophilus* DGCC7710, which is sensitive to *cos*- (D5691, D5913, D6037 and D6215) and *pac*-type phages (858, 2972, D3288, 4259, D4752, D4754 and D939) (Table 2). Finally, the mutation was introduced into *S*. *thermophilus* DGCC7796, which is also sensitive to *cos*- (D4090, D5821 and D4807) and *pac-*phages (D2765, D4274, D5787 and M5876) (Table 2). Although the mutation was less efficient against all four *cos*-type phages infecting *S*. *thermophilus* DGCC7710 (EOP 10^−4^), the mutation provided resistance against all *cos*-type phages tested. The introduction of MetAP^H206Q^ into *S*. *thermophilus* DGCC7796 and DGCC782 led to a reduction in the EOP of at least 10^−6^ for all *cos-*type phages (Table 2), which corresponded to the limit of detection. No phage mutant could be recovered from these assays. However, none of the *S*. *thermophilus pac*-type phages were affected by the mutation, suggesting that MetAP is not key for the replication of these phages.

To determine if the MetAP^H206Q^ mutation would provide resistance to another streptococcal species, we introduced it into the *S*. *mutans* strain HER 1503, which is sensitive to the *cos*-type virulent phage M102AD^43^. The methionine aminopeptidase gene of *S*. *mutans* HER 1503 has the same length (861 bp) as that of *S*. *thermophilus*. The two genes share 74% identity while the two MetAP share 85% identity. The histidine residue found at position 206 is present in *S*. *mutans*. The MetAP^H206Q^ mutation inserted into *S*. *mutans* provided complete resistance to phage M102AD (EOP <1 × 10^−7^). Complementation of the mutated strain with the wild-type *metAP* restored phage sensitivity. Our data suggest a relatively widespread role of MetAP in phage replication.

### Other mutations in MetAP affect replication of phage DT1

To determine if other mutations in the *metAP* gene provide phage resistance, we used an error-prone PCR amplification approach. The resulting PCR products were transformed into *S*. *thermophilus* SMQ-301 using natural competence. Since we were not introducing an antibiotic resistance marker, we used phage DT1 to select the clones. The transformation assay with the positive control (MetAP^H206Q^ mutation) resulted in many more BIMs than with the negative control (no DNA) (Fig. S1). Fewer BIMs were obtained with the PCR product containing random mutations than with the positive control (Fig. S1). The *metAP* gene was sequenced in 10 randomly selected clones and all of them had various mutations. Seven mutants had a single mutation in the *metAP* gene resulting in an amino acid substitution. We tested phage sensitivity of these mutants and they were all resistant to phage DT1. We complemented the 10 resistant strains with pNZ123:metAP and as previously observed, the wild-type MetAP restored phage sensitivity, confirming that other mutations in the MetAP can confer phage resistance (Table 4; Fig. 1).

**Table 4:** Random mutagenesis of the *metAP* gene of *S*. *thermophilus* SMQ-301 and resistance to DT1.

Name	Position in the gene	Mutation	Codon change	Amino acid change	EOP	EOP after complementation
MetAP_S7	205	C > A	CAG > AAG	Q57K	<×10^−6^	5 × 10^−1^
MetAP_S11	538	C > A	GCG > GAG	A168E	<×10^−6^	3 × 10^−3^
MetAP_S14	523	T > C	TAT > TAC	Y162Y	<×10^−6^	5 × 10^−2^
	657	A > G	GAG > GGA	E207G		
	660	A > G	GAG > GGA	E208G		
MetAP_S21	711	G > T	GGA > TGA	G226X	<×10^−6^	3 × 10^−1^
MetAP_S32	698	C > A	CCA > CAA	P233Q	<×10^−6^	7 × 10^−1^
MetAP_S36	698	C > T	CCA > CTA	P233L	<×10^−6^	6 × 10^−2^
MetAP_S43	37	A > G	ATG > GTG	M13V	<×10^−6^	2 × 10^−1^
	240	A > G	GCA > GCG	A80A		
	484	T > G	TAT > GAT	Y162D		
MetAP_S44	600	A > G	GGA > GGG	G200G	<×10^−6^	1.0
	618	G > A	CAG > CAA	H206Q		
MetAP_S45	458	T > C	CTT > CCT	L153P	<×10^−6^	1.1
MetAP_R22	684	T > A	GTC > GAC	V228D	<×10^−6^	1.0

### Phage adsorption and phage DNA replication are not affected

**Figure 2:**
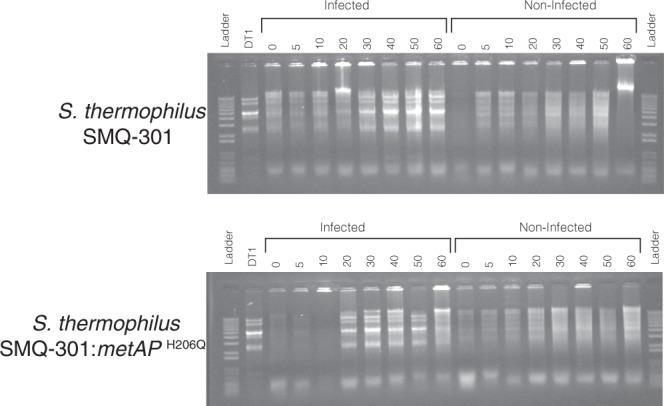
DNA replication of DT1 in the wild-type *S*. *thermophilus* SMQ-301 and *S*. *thermophilus* SMQ-301:metAP^H206Q^. Values above electrophoresis gels represent time points (in min) at which total DNA was extracted from cells grown in the presence or absence of phage DT1.

To understand the molecular mechanism underlying the phage resistance provided by this mutation, we dissected the lytic cycle of a phage infecting a mutated strain. First, we tested if phage DT1 could still adsorb to *S*. *thermophilus* SMQ-301:metAP^H206Q^ (Table 3). The viral particles adsorbed at a slightly higher level on the wild-type phage-sensitive host (93%) than on the spontaneous BIMs #2, #3, and #5 (80% to 86%). The adsorption of DT1 was more reduced on *S*. *thermophilus* SMQ-301:metAP^H206Q^ (68%). However, this reduced adsorption cannot explain the strong phage resistance phenotype (EOP of 10^−8^), suggesting that another step of the lytic cycle is impeded by the MetAP^H206Q^ mutation. Since phage DT1 can adsorb to the BIM surface, we tested whether phage DNA was replicated inside the bacterial cell. Both *S*. *thermophilus* SMQ-301 and SMQ-301:metAP^H206Q^ were infected with phage DT1 using a MOI of 5 and total DNA was extracted from the infected cells at different time points, digested with a restriction enzyme and migrated on an agarose gel (Fig. 2). The DNA fragments corresponding to DT1 digested genome were visible after 20 min of infection in both the MetAP^H206Q^ mutant and the wild-type cells, indicating that the phage DNA entered the bacterial cell and was replicated (Fig. 2). Lysis of the culture was only observed with *S*. *thermophilus* SMQ-301.

### The MetAP^H206Q^ mutation affects N-terminal methionine processing

**Figure 3:**
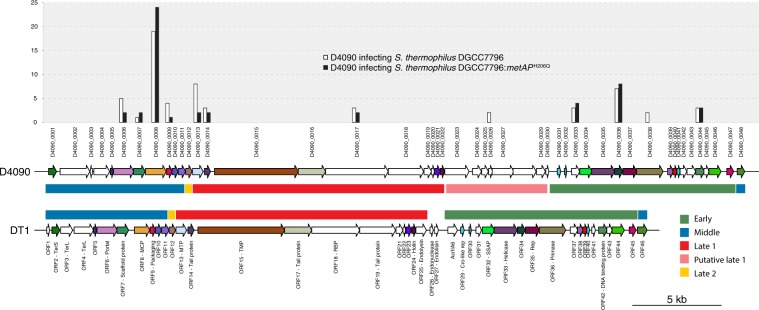
Genetic alignment of phages D4090 and DT1. Each protein-coding gene is represented by an arrow. When two deduced proteins share 70% identity or more, they are represented with the same color, otherwise they are shown in white. The boxes between the two genomes represent the expression modules. Phage D4090 expression modules were extrapolated from DT1 experimental data. The graph above the alignment represents the relative abundance of the proteins when phage D4090 infects the wild-type strain DGCC7796 and mutant DGCC7796:metAP^H206Q^.

Since the MetAP^H206Q^ mutation does not affect phage DNA replication, we investigated whether protein expression was affected by the mutation. To compare the activity of MetAP, we used *S. thermophilus* DGCC7796, a strain sensitive to *cos*- and *pac*-type phages. Using mass spectrometry (LC-MS/MS), we analyzed the complete proteome of the wild-type and mutant DGCC7796 after 20 min of infection with phages D4090 (*cos*-type) or M5876 (*pac*-type) using a MOI of 5. During the replication of phage D4090, although we observed a slight difference in protein expression, there was no significant trend in protein expression between mutant and wild-type strains (Fig. 3, Supp. Table 1). Peptides of 10 and 12 phage proteins were detected in the wild-type and mutant strains, respectively, and their relative abundance was similar in both strains. The difference in the number of proteins detected can be explained by the very low level of detection of the corresponding proteins in the wild-type strain.

**Figure 4:**
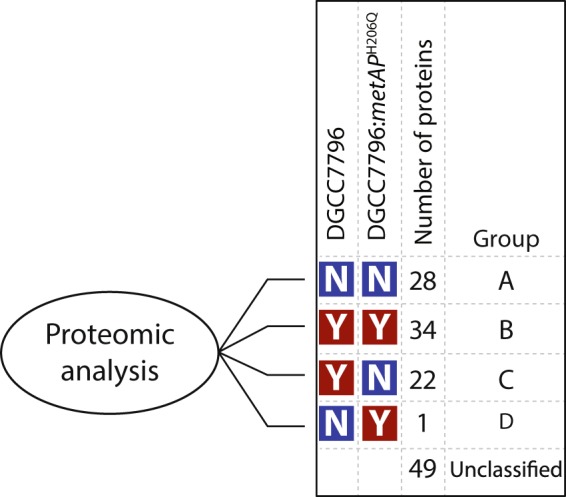
Proteomic analysis of the N-terminal peptides from the proteome of the wild-type strain DGCC7796 and mutant DGCC7796:metAP^H206Q^. Post-translational processing of the N-terminal methionine was detected in both proteomes but it was less abundant in the mutant strain. N represents unprocessed peptides while Y stands for processed peptides.

We then used the proteomic datasets to verify the activity of the mutant and wild-type MetAP. We generated two distinct databases of peptides to search against. The first database was composed of only N-terminal peptides after tryptic digestion. The second database was essentially the same as the first one but we removed the N-terminal methionine of each peptide. This allowed us to determine if the protein underwent N-terminal post-translational processing. The complete database included the 2,051 proteins encoded by the host genome and the 41 proteins encoded by the phage genome. Following in silico tryptic digestion, the N-terminal peptide database included 579 peptides of 8–30 amino acids that have a molecular mass that could be detected with the setting used to conduct the mass spectrometry analysis. Using LC-MS/MS, we detected a total of 134 from 579 N-terminal peptides across all samples analyzed (Table S2). We did not observe any difference in post-translational processing of the N-terminal methionine between the uninfected and infected samples nor between the *cos-* and *pac-*type phage-infected wild-type cells, suggesting that phage infection does not affect MetAP activity. Then, we compared the data with the MetAP mutated cells and classified the results into four groups: A) 28 proteins were unprocessed by MetAP in both wild-type and mutant strains, B) 34 proteins were processed by MetAP in both the wild-type and mutant strains, C) 22 proteins were processed by MetAP in the wild-type strain only, and D) only one protein was processed by MetAP in the mutated strain (Fig. 4).

*E. coli* methionine aminopeptidase is more specific for protein with a penultimate amino acid (a.a.) with small side chain (e.g. Ala, Gly, Pro, Ser, Thr, or Val)^49,50^. Thus, it is not surprising that most proteins (Thr is the only exception out of 28) found in group A (non-processed) have penultimate a.a. with long side chain (Ile, Asn, Leu, Glu, Tyr, Gln and Asp). Most of the penultimate a.a. of proteins found in groups B and C have short side chain (Ala, Gly, Pro, Ser, Thr, or Val) with only two exceptions found in group C (Cys and Lys). Since there are no significant difference in the penultimate a.a. of groups B and C, the findings that some proteins were not processed by the mutated MetAP (group C) suggest that its activity is impaired to some degree. Analyzing functions of the above proteins and metabolic pathways in which they are involved did not reveal why phage infection was blocked in presence of the mutated MetAP (Supp. Table 2).

### The MetAP^H206Q^ mutation affects growth of the bacterial strains

**Figure 5:**
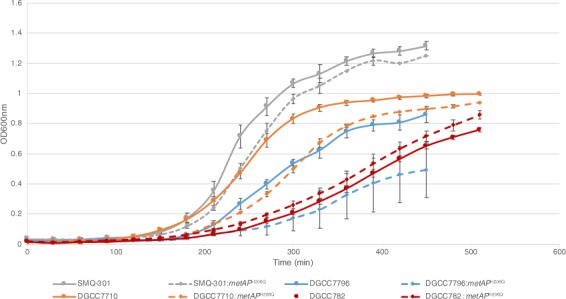
Growth curves of the wildtype and mutant *S*. *thermophilus* strains.

Growth of the mutant strains was also evaluated (Fig. 5). Surprisingly, the MetAP^H206Q^ mutation had different impact depending of the *S*. *thermophilus* strains. The growth rate difference between the wild-type strains and their mutants was not significant, except for *S*. *thermophilus* DGCC7796 and its mutant for which the growth rates were respectively 36.2 ± 3.6 min and 53.5 ± 3.3 min (Table 5). We also observed a latent period longer with *S*. *thermophilus* DGCC7796:metAP^H206Q^ than with the other strains tested (Fig. 5).

**Table 5:** Generation time of *S*. *thermophilus* wild-type and mutant strains.

*S*. *thermophilus* strain	Generation time (min)
SMQ-301	36.7 ± 1.8
SMQ-301:metAP^H206Q^	38.1 ± 1.1
DGCC7796	36.2 ± 3.6
DGCC7796:metAP^H206Q^	53.5 ± 3.3
DGCC7710	38.1 ± 2.1
DGCC7710:metAP^H206Q^	38.6 ± 1.7
DGCC782	49.7 ± 1.8
DGCC782:metAP^H206Q^	53.4 ± 4.4

### The MetAP^H206Q^ mutation is stable

One of the important features for industrial fermentation is the stability of the phage resistance phenotype. Thus, we verified the stability of the mutation MetAP^H206Q^ over 60 generations of *S*. *thermophilus* SMQ-301 and DGCC7796 as well as their mutants in milk and LM17. While all selected colonies of the wild -type strains remained phage sensitive, all 10 colonies from each mutant remained phage resistant, indicating that the MetAP^H206Q^ mutation is stable.

## Conclusion

While cell wall proteins and polysaccharides were identified as host factors needed for phage infection, the literature is sparse about cytoplasmic proteins. MetAP are cytoplasmic enzymes found in all living organisms^51^. They are a unique class of proteases that remove the N-terminal residue from nascent proteins and play a central role in the synthesis and maturation of proteins in prokaryotes and eukaryotes^52^. The essentiality of this post-translational processing is underscored by the lethality of *metAP* gene inactivation in *E*. *coli*^53^, *Salmonella typhimurium*^54^, and *Saccharomyces cerevisiae*^55^. This dependence now extends to some of their parasites.

In this study, we showed that phage resistance can be acquired through mutations in the *metAP* gene of *S*. *thermophilus* and *S*. *mutans*. We were unable to isolate phage mutants that overcame MetAP^H206Q^ mutation, confirming that such mutation can provide a robust phage resistance. Early steps (adsorption, DNA replication, protein expression) of the phage lytic cycle occurred in phage-infected *S*. *thermophilus* SMQ-301:metAP^H206Q^, suggesting that this mutation acts lately during the lytic cycle, perhaps at the virion assembly step. Since the catalytic activity of MetAP is at the core of living cells, we expected that the MetAP^H206Q^ mutation provided a broad phage resistance. Although the replication of *cos*-type phages was affected by MetAP mutation, the *pac*-type phages were not. As the enzymatic activity of MetAP:H206Q was less effective than the wild-type MetAP, it appears that *cos*-type phages require efficient cleavage of the N-terminal methionine of some of their proteins and/or of their host to complete their lytic cycle.

## Supplementary information

Supplementary materials
