# Author Correction: A mutation in the methionine aminopeptidase gene provides phage resistance in *Streptococcus thermophilus*

**DOI:** 10.1038/s41598-020-59767-w

**Published:** 2020-02-11

**Authors:** Simon J. Labrie, Cas Mosterd, Stéphanie Loignon, Marie-Ève Dupuis, Philippe Desjardins, Geneviève M. Rousseau, Denise M. Tremblay, Dennis A. Romero, Philippe Horvath, Christophe Fremaux, Sylvain Moineau

**Affiliations:** 1 Département de biochimie, de microbiologie, et de bio-informatique, Faculté des sciences et de genie, Université Laval, Québec City, QC G1V 0A6 Canada; 2 Groupe de recherche en écologie buccale, Faculté de médecine dentaire, Université Laval, Québec City, QC G1V 0A6 Canada; 3 Félix d’Hérelle Reference Center for Bacterial Viruses, Faculté de médecine dentaire, Université Laval, Québec City, QC G1V 0A6 Canada; 4 DuPont Nutrition and Biosciences, 3329 Agriculture Dr, Madison, WI 53716 USA; 5 DuPont Nutrition and Biosciences, BP10, Dangé-Saint-Romain, 86220 France; 6 Present Address: SyntBioLab Inc., 4820-250, rue de la Pascaline, Lévis, G6W 0L9 Canada

Correction to: *Scientific Reports* 10.1038/s41598-019-49975-4, published online 25 September 2019

This Article contains errors in Table 4. In the HTML and PDF versions of this Article, the positions of the mutation in the gene are inaccurate. The correct Table 4 appears below.

**Table 4:** Random mutagenesis of the *metAP* gene of *S. thermophilus* SMQ301 and resistance to DT1.

Name	Position in the gene	Mutation	Codon change	Amino acid change	EOP	EOP after complementation
MetAP_S7	169	C>A	CAG>AAG	Q57K	<×10^−6^	5 × 10^−1^
MetAP_S11	503	C>A	GCG>GAG	A168E	<×10^−6^	3 × 10^−3^
MetAP_S14	486	T>C	TAT>TAC	Y162Y	<×10^−6^	5 × 10^−2^
	620	A>G	GAG>GGA	E207G		
	623	A>G	GAG>GGA	E208G		
MetAP_S21	676	G>T	GGA>TGA	G226X	<×10^−6^	3 × 10^−1^
MetAP_S32	698	C>A	CCA>CAA	P233Q	<×10^−6^	7 × 10^−1^
MetAP_S36	698	C>T	CCA>CTA	P233L	<×10^−6^	6 × 10^−2^
MetAP_S43	37	A>G	ATG>GTG	M13V	<×10^−6^	2 × 10^−1^
	240	A>G	GCA>GCG	A80A		
	484	T>G	TAT>GAT	Y162D		
MetAP_S44	600	A>G	GGA>GGG	G200G	<×10^−6^	1.0
	618	T>A	CAT>CAA	H206Q		
MetAP_S45	458	T>C	CTT>CCT	L153P	<×10^−6^	1.1
MetAP_R22	683	T>A	GTC>GAC	V228D	<×10^−6^	1.0

