# Supplementary material for: A mutation in the methionine aminopeptidase gene provides phage resistance in Streptococcus thermophilus
Source: Sci Rep. 2019 Sep 25;9:13816. doi: 10.1038/s41598-019-49975-4 (PMC6761271; doi:10.1038/s41598-019-49975-4)

A mutation in the methionine aminopeptidase gene provides phage resistance in *Streptococcus thermophilus*

Simon J. Labrie[1,2†], Cas Mosterd[1,2], Stéphanie Loignon[2,3], Marie-Ève Dupuis[1,2], Philippe Desjardins[1], Geneviève M. Rousseau[1,2], Denise M. Tremblay[2,3] Dennis A. Romero[4], Philippe Horvath[5], Christophe Fremaux[5], and Sylvain Moineau[1,2,3*]

[1] Département de biochimie, de microbiologie, et de bio-informatique, Faculté des sciences et de génie, Université Laval, Québec City, QC, Canada, G1V 0A6

[2] Groupe de recherche en écologie buccale, Faculté de médecine dentaire, Université Laval, Québec City, QC, Canada, G1V 0A6

[3] Félix d'Hérelle Reference Center for Bacterial Viruses, Faculté de médecine dentaire, Université Laval, Québec City, QC, Canada, G1V 0A6

[4] DuPont Nutrition and Health, 3329 Agriculture Dr, Madison, WI 53716, USA

[5] DuPont Nutrition and Health, BP10, Dangé-Saint-Romain 86220, France

†Current address: SyntBioLab Inc. 4820, rue de la Pascaline, Lévis, Qc, Suite 250, G6W 0L9

* To whom correspondence should be addressed. Tel: +1 418 656 3712,

Email: Sylvain.Moineau@bcm.ulaval.ca

Table S1. List of primers

Primer	Sequence 5’-3’	Function	Ref
SJL128	TCAATCTACTCAAGGTATGAATCA	Natural transformation of *metAP*	This study
SJL130	GTCAGTAGTAGTGGTCAAGA	Natural transformation of *metAP*	This study
SJL150	AGTTCCTGATAGGTCGCATT	Detection of the mutation in *metAP*	This study
SJL151	GAGTTGGACCAACAATGCAG	Detection of the mutation in *metAP*	This study
SJL154	CAGCAGCGGCCTGGTGCCGCGCGGCAGCCAAATGAT TACACTGAAATCAGCACGTG	Cloning *metAP* in pNZ123	This study
SJL155	GCCGGATCTCAGTGGTGGTGGTGGTGGTGCTTAATAA GTTCTTTCTTCCCCTTGAG	Cloning *metAP* in pNZ123	This study
SJL160	ATTACAGCTCCAGATCCAGTACTGAATTCTTGAGCCT GCTATGATTGACTCTGCA	Cloning the mutated *S. mutans metAP* in pNZ123	This study
SJL161	ATTGGGTTCTTCCTGCATGGTTGG	Cloning the mutated *S. mutans metAP* in pNZ123	This study
SJL162	CCAACCATGCAGGAAGAACCCAAT	Cloning the mutated *S. mutans metAP* in pNZ123	This study
SJL163	GAAAATATGCACTCGAGAAGCTTGAGCTCTCCGAAG GTGGACAACATAATAGC	Cloning the mutated *S. mutans metAP* in pNZ123	This study
CM_145	GAATTCGAATTCAAAGGCTGTTGTGACAGCAA	Cloning the *S. mutans metAP* in pNZ123	This study
CM_146	CTCGAGCTCGAGTTAATAAGTCCCTTCTTGACCC	Cloning the *S. mutans metAP* in pNZ123	This study
CR1-fwd	TGCTGAGACAACCTAGTCTCTC	CR1 locus screening	32
CR1-revLong	TAAACAGAGCCTCCCTATCC	CR1 locus screening	32
CR3-fwd	CTGAGATTAATAGTGCGATTACG	CR3 locus screening	32
CR3-rev	GCTGGATATTCGTATAACATGTC	CR3 locus screening	32

Underlined nucleotides correspond to either restriction sites or part of the primer sequence complementary to the cloning vector.

Table S2. Analysis of the post-translational processing of the N-terminal methionine

Group	Accession.Number	Putative.function	TIGR_Fam	Link	Role	SubRole	DGCC7796	DGCC7796 + D4090	DGCC7796:MetAP H206Q	DGCC7796:MetAP H206Q + D4090	DGCC7796 + M5876	DGCC7796:MetAP H206Q + M5876
A	DGCC7796_0002	DNA polymerase III beta subunit	TIGR00663	132	DNA metabolism	Degradation of DNA	N	N	N	N	N	N
A	DGCC7796_0312	COG1399 protein in cluster with ribosomal protein L32p	TIGR00092	157	Unknown function	Conserved			N		N	N
A	DGCC7796_0406	1-phosphofructokinase					N	N	N	N	N	
A	DGCC7796_0563	Mercuric ion reductase					N	N	N	N	N	N
A	DGCC7796_0581	Threonyl-tRNA synthetase	TIGR00745	83	Biosynthesis of cofactors, prosthetic groups, and carriers	Molybdopterin			N		N	N
A	DGCC7796_0620							N		N		
A	DGCC7796_0641	Catabolite control protein A	TIGR00115	95	Protein fate	Toxin production and resistance		N	N		N	N
A	DGCC7796_0702	2-haloalkanoic acid dehalogenase						N	N		N	
A	DGCC7796_1159							N		N	N	N
A	DGCC7796_1159	LSU ribosomal protein L19p	TIGR01979	76	Biosynthesis of cofactors, prosthetic groups, and carriers	Serine family	N	N	N	N	N	N
A	DGCC7796_1250	FIG137478: Hypothetical protein YbgI						N	N		N	N
A	DGCC7796_1295	Phenylalanyl-tRNA synthetase alpha chain					N	N	N	N		
A	DGCC7796_1361	oxidoreductase of aldo/keto reductase family, subgroup 1					N	N	N	N	N	N
A	DGCC7796_1424							N		N		
A	DGCC7796_1657	Thioredoxin reductase					N	N	N	N	N	N
A	DGCC7796_1741	Sucrose-6-phosphate hydrolase (EC 3.2.1.B3)	TIGR00040	703	Unknown function	Conjugation	N	N	N	N	N	N
A	DGCC7796_1757	Single-stranded DNA-binding protein					N	N	N	N	N	N
A	DGCC7796_1861	Pyrroline-5-carboxylate reductase	TIGR00232	117	Energy metabolism	Glycolysis/gluconeogenesis	N	N	N	N	N	
A	DGCC7796_1880	Tyrosyl-tRNA synthetase					N	N	N	N		N
A	DGCC7796_1905	Hypothetical protein ywlG							N		N	
A	DGCC7796_1922	hypothetical protein	TIGR01136	75	Amino acid biosynthesis	Pyruvate family	N	N	N	N		N
A	DGCC7796_1932							N		N		
A	DGCC7796_1956	Preprotein translocase secY subunit					N	N	N	N	N	N
A	DGCC7796_2046	ABC transporter ATP-binding protein uup	TIGR03168					N	N			
A	DGCC7796_0282										N	N
A	M5876_0033		TIGR00061	158	Protein synthesis	General					N	N
A	M5876_0036		TIGR00496	169	Protein synthesis	tRNA and rRNA base modification					N	N
A	DGCC7796_1173										N	N
B	DGCC7796_0004	GTP-binding and nucleic acid-binding protein YchF							Y		Y	Y
B	DGCC7796_0034							Y		Y		
B	DGCC7796_0072		TIGR00962	111	Energy metabolism	Anaerobic		Y		Y		
B	DGCC7796_0093	2-dehydropantoate 2-reductase	TIGR01216	111	Energy metabolism	Anaerobic	Y	Y	Y	Y	Y	Y
B	DGCC7796_0117							Y		Y		
B	DGCC7796_0129	Cell division trigger factor	TIGR00855	158	Protein synthesis	General	Y	Y	N	Y	Y	Y
B	DGCC7796_0229	Aminopeptidase C	TIGR01891	138	Protein fate	tRNA aminoacylation	Y	Y	Y	N	Y	Y
B	DGCC7796_0242							Y		Y		
B	DGCC7796_0366	Cysteine synthase	TIGR01173	89	Cell envelope	Other	Y	N	N	N	Y	Y
B	DGCC7796_0371		TIGR00418	137	Protein synthesis	Other		Y		Y		
B	DGCC7796_0392		TIGR01123	74	Amino acid biosynthesis	Glutamate family		Y		Y		
B	DGCC7796_0460							Y		Y		
B	DGCC7796_0481							Y		Y		
B	DGCC7796_0486	ATP synthase alpha chain	TIGR01481	261	Regulatory functions	Chlorophyll and bacteriochlorophyll	Y	Y	Y	Y	Y	Y
B	DGCC7796_0489	ATP synthase epsilon chain	TIGR01060	116	Energy metabolism	Fermentation		Y			Y	Y
B	DGCC7796_0542	LSU ribosomal protein L7/L12 (P1/P2)	TIGR00499	137	Protein synthesis	Other	Y	Y	Y	Y	Y	Y
B	DGCC7796_0648	Enolase	TIGR01549	703	Unknown function	Conjugation	Y	Y	Y	Y	Y	Y
B	DGCC7796_0747	Cell division protein FtsZ	TIGR00065	93	Cellular processes	Other		Y	Y		Y	Y
B	DGCC7796_0752	Cell division initiation protein DivIVA	TIGR03544				N	Y	Y	N	Y	Y
B	DGCC7796_0837	Tripeptide aminopeptidase					Y		Y		Y	Y
B	DGCC7796_0982	Orotate phosphoribosyltransferase					Y	Y	Y		Y	Y
B	DGCC7796_1299	Aspartate-semialdehyde dehydrogenase	TIGR00536	140	Protein fate	Degradation of proteins, peptides, and glycopeptides	Y	Y		Y	Y	Y
B	DGCC7796_1359	NADH dehydrogenase	TIGR01882	138	Protein fate	tRNA aminoacylation	Y	Y	Y	Y		N
B	DGCC7796_1399	UDP-glucose 4-epimerase	TIGR01886	138	Protein fate	tRNA aminoacylation	Y	Y	Y	Y	Y	Y
B	DGCC7796_1624	Nicotinate-nucleotide adenylyltransferase	TIGR00107	127	Purines, pyrimidines, nucleosides, and nucleotides	Pyrimidine ribonucleotide biosynthesis			Y		Y	Y
B	DGCC7796_1782	Glutamine synthetase type I					Y	Y	Y	Y	Y	Y
B	DGCC7796_1797	SSU ribosomal protein S12p (S23e)	TIGR02093	105	Energy metabolism	Polyamine biosynthesis	Y	Y	Y	Y		
B	DGCC7796_1881	Ketol-acid reductoisomerase	TIGR00972	143	Transport and binding proteins	Amino acids, peptides and amines	Y	Y	Y	Y	Y	Y
B	DGCC7796_1940		TIGR00336	126	Purines, pyrimidines, nucleosides, and nucleotides	Purine ribonucleotide biosynthesis		Y		Y		
B	DGCC7796_1941		TIGR01870	719				Y		Y		
B	DGCC7796_1944	SSU ribosomal protein S8p (S15Ae)					Y	Y	Y	Y	Y	Y
B	DGCC7796_0921 (+1)										Y	Y
B	DGCC7796_1890										Y	Y
B	DGCC7796_1987		TIGR00884	125	Purines, pyrimidines, nucleosides, and nucleotides	Nucleotide and nucleoside interconversions					Y	Y
C	DGCC7796_0201		TIGR00686	157	Unknown function	Conserved		Y		N		
C	DGCC7796_0257	phosphoesterase	TIGR01135	89	Cell envelope	Other	Y	Y	N	N	Y	N
C	DGCC7796_0308	Transketolase					Y	Y	N	Y	Y	N
C	DGCC7796_0331	FIG01115503: hypothetical protein						Y	N			N
C	DGCC7796_0409		TIGR01024	158	Protein synthesis	General		Y		N		
C	DGCC7796_0602	Branched-chain amino acid aminotransferase	TIGR02651	166	Transcription	Transcription factors	Y	Y	N	N	Y	
C	DGCC7796_0759	3'-to-5' oligoribonuclease A					Y	Y	N	N	Y	N
C	DGCC7796_0848	Acetylornithine deacetylase/Succinyl-diaminopimelate desuccinylase						Y	N	N	Y	N
C	DGCC7796_0941	Maltodextrin phosphorylase	TIGR00486	157	Unknown function	Conserved	Y	Y	N	N	Y	N
C	DGCC7796_1052	GMP synthase [glutamine-hydrolyzing], ATP pyrophosphatase subunit					Y	Y	N	N	Y	N
C	DGCC7796_1064	Glucosamine--fructose-6-phosphate aminotransferase [isomerizing]						Y	N		Y	
C	DGCC7796_1214	Cystathionine beta-lyase	TIGR00468	137	Protein synthesis	Other	Y	Y	N	N	Y	
C	DGCC7796_1410	Glutathione biosynthesis bifunctional protein gshF	TIGR01296	71	Amino acid biosynthesis	Other	Y		N	N	Y	N
C	DGCC7796_1525	Phosphoserine aminotransferase					Y	Y	N	N	Y	N
C	DGCC7796_1643	Universal stress protein family							N		Y	N
C	DGCC7796_1714	Gamma-glutamyl phosphate reductase	TIGR01179	119	Energy metabolism	Pyruvate dehydrogenase	Y		N			
C	DGCC7796_1744	Alkaline shock protein	TIGR01435	86	Biosynthesis of cofactors, prosthetic groups, and carriers	Riboflavin, FMN, and FAD	Y	Y	N	N	Y	N
C	DGCC7796_1813	putative transport accessory protein					Y	Y	N		Y	N
C	DGCC7796_1939							Y		N		
C	DGCC7796_1964	Adenylosuccinate synthetase	TIGR01214	90	Cell envelope	Biosynthesis and degradation of murein sacculus and peptidoglycan	Y	Y		N	Y	
C	DGCC7796_2024		TIGR01364	75	Amino acid biosynthesis	Pyruvate family		Y		N		N
C	DGCC7796_0760										Y	N
D	DGCC7796_0699	Lysyl-tRNA synthetase (class II)	TIGR00125				N		Y			
P	DGCC7796_1476	dTDP-4-dehydrorhamnose reductase					N	N	N	N	Y	N
P	DGCC7796_1828	Carbonic anhydrase	TIGR01292	112	Energy metabolism	ATP-proton motive force interconversion	Y	Y	N	N	Y	Y
P	DGCC7796_1937	Adenylate kinase	TIGR00407	73	Amino acid biosynthesis	Aspartate family	N	N	N	N	Y	N
U	DGCC7796_0130	DNA-directed RNA polymerase delta subunit	TIGR01322	105	Energy metabolism	Polyamine biosynthesis	N	N	N	N	Y	N
	DGCC7796_0155	Amino acid transport ATP-binding protein							N			
	DGCC7796_0162	Cysteine desulfurase, SufS subfamily	TIGR00621	132	DNA metabolism	Degradation of DNA			N			
	DGCC7796_0202		TIGR00653	73	Amino acid biosynthesis	Aspartate family		Y		Y		
	DGCC7796_0236							Y				
	DGCC7796_0302	Methionine ABC transporter permease protein	TIGR00981	158	Protein synthesis	General			N			
	DGCC7796_0417	LSU ribosomal protein L21p							N			N
	DGCC7796_0440	Ribosome recycling factor							N	N		N
	DGCC7796_0541	LSU ribosomal protein L10p (P0)							N	N		N
	DGCC7796_0560	Aminoacylase/N-acyl-L-amino acid amidohydrolase/hippurate hydrolase	TIGR00112	73	Amino acid biosynthesis	Aspartate family			N	N		N
	DGCC7796_0570	Glucosamine-1-phosphate N-acetyltransferase	TIGR00234	137	Protein synthesis	Other	Y	Y			Y	
	DGCC7796_0607		TIGR00465	74	Amino acid biosynthesis	Glutamate family		Y				
	DGCC7796_0753		TIGR01440	156	Hypothetical proteins	Transposon functions				N		
	DGCC7796_0764	Protein-N(5)-glutamine methyltransferase PrmC							N			
	DGCC7796_0861	Purine nucleoside phosphorylase	TIGR00564	70	Amino acid biosynthesis	Other				N		
	DGCC7796_0921	Dihydrolipoamide acetyltransferase	TIGR00167	116	Energy metabolism	Fermentation	Y	Y	N	Y		
	DGCC7796_0948	Phosphate transport ATP-binding protein PstB							N		Y	
	DGCC7796_0988	CRISPR-associated protein, Csm2 family	TIGR01351	124	Purines, pyrimidines, nucleosides, and nucleotides	2'-Deoxyribonucleotide metabolism			N			
	DGCC7796_1002	Fibronectin/fibrinogen-binding protein							N			
	DGCC7796_1032	FIG01119309: hypothetical protein							N			
	DGCC7796_1037							Y				
	DGCC7796_1063	Alkylphosphonate utilization operon protein PhnA							N			N
	DGCC7796_1147	Phosphoglucomutase							N	N		N
	DGCC7796_1213	Ribonuclease Z								N		
	DGCC7796_1245	dTDP-4-dehydrorhamnose 3,5-epimerase								N		N
	DGCC7796_1267							Y				
	DGCC7796_1272							Y				
	DGCC7796_1469	rhamnose-containing polysacharide translocation permease							N			
	DGCC7796_1535	Putrescine transport ATP-binding protein PotA	TIGR00184	125	Purines, pyrimidines, nucleosides, and nucleotides	Nucleotide and nucleoside interconversions		Y				
	DGCC7796_1794							Y				
	DGCC7796_1814							N				
	DGCC7796_1917	hypothetical protein								N		
	DGCC7796_1925	Fructose-bisphosphate aldolase class II							N	N		N
	DGCC7796_1942							Y				
	DGCC7796_1947							N				
	DGCC7796_1952							N				
	DGCC7796_1953									N		N
	DGCC7796_1955							Y				
	DGCC7796_0031											N
	DGCC7796_0095											N
	DGCC7796_0330										y	
	DGCC7796_0388											N
	DGCC7796_0626											N
	DGCC7796_0649											N
	DGCC7796_1461											N
	M5876_0027										N	

Y : processed
N : not processed
Groups A-D refer to the classification of Figure 4.

Figure S1. Frequency of the BIMs naturally transformed A) without DNA, B) with MetAP^{H206Q} or C) with a MetAP obtain following an error prone PCR.

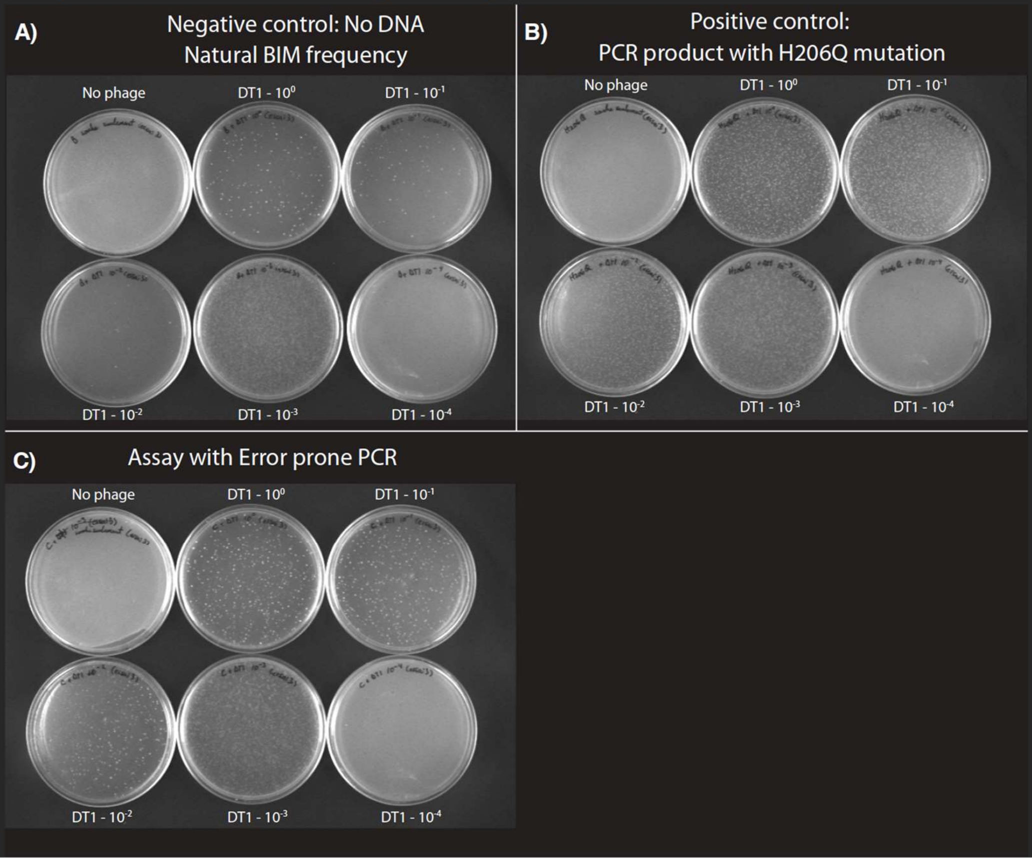

Supplement: Supplementary file 1 — Supplementary materials [file 41598_2019_49975_MOESM1_ESM.pdf]
